# Co-Morbidity, Mortality, Quality of Life and the Healthcare/Welfare/Social Costs of Disordered Sleep: A Rapid Review

**DOI:** 10.3390/ijerph13080831

**Published:** 2016-08-18

**Authors:** Sergio Garbarino, Paola Lanteri, Paolo Durando, Nicola Magnavita, Walter G. Sannita

**Affiliations:** 1 Center of Sleep Medicine, Genoa 16132, Italy; sgarbarino.neuro@gmail.com (S.G.); wgs@dism.unige.it (W.G.S.); 2 Department of Neuroscience, Ophthalmology, Genetics, Maternal and Child Health, University of Genoa, Genoa 16132, Italy; 3 Child Neurology and Psychiatry Unit, Istituto Giannina Gaslini, Genoa 16148, Italy; paolanteri@yahoo.it; 4 Department of Health Sciences, Postgraduate School in Occupational Medicine, University of Genoa and Occupational Medicine Unit, IRCCS AOU San Martino IST, Genoa 16132, Italy; paolo.durando@unige.it; 5 Department of Public Health, Università Cattolica del Sacro Cuore, Rome 00168, Italy

**Keywords:** sleep disorders, quality of life, public health, mortality, morbidity, cardiovascular disorders, cancer, accidents

## Abstract

Sleep disorders are frequent (18%–23%) and constitute a major risk factor for psychiatric, cardiovascular, metabolic or hormonal co-morbidity and mortality. Low social status or income, unemployment, life events such as divorce, negative lifestyle habits, and professional requirements (e.g., shift work) are often associated with sleep problems. Sleep disorders affect the quality of life and impair both professional and non-professional activities. Excessive daytime drowsiness resulting from sleep disorders impairs efficiency and safety at work or on the road, and increases the risk of accidents. Poor sleep (either professional or voluntary) has detrimental effects comparable to those of major sleep disorders, but is often neglected. The high incidence and direct/indirect healthcare and welfare costs of sleep disorders and poor sleep currently constitute a major medical problem. Investigation, monitoring and strategies are needed in order to prevent/reduce the effects of these disorders.

## 1. Introduction

Sleep is necessary to maintain a physiological/psychological equilibrium and for homeostatic adaptation. Sleep disorders are often associated with (or add to the risk of) psychiatric, cardiovascular, metabolic or hormonal diseases [1]. Poor sleepers complain of mood changes, cognitive impairment, increased drowsiness, anxiety, fatigue or reduced pain tolerance during the day more often than good sleepers. Sleep disorders affect personal, family or social lives as well as impairing professional efficiency and increasing the risk of accidents and health and welfare costs [2]. Personal or social factors such as unemployment, family problems such as divorce, low income, and negative lifestyles increase the incidence of sleep problems and enhance their effects on everyday life. Sleep disorders and insufficient sleep duration seem to be endemic in our contemporary society (about 18% in Europe and 23% in the U.S.) [3] and currently constitute a health, welfare and social problem that requires close monitoring and prevention [4].

An analysis of the latest literature using a method that simplifies the components of a systematic review [5] formed the basis of the critical review of evidence presented in this study. This rapid review was completed between March and April 2016.

## 2. Effects of Sleep Disorders

### 2.1. Morbidity and Co-Morbidity

Surveys and population-based studies record higher co-morbidity among subjects with sleep problems [6,7,8]. Sleep breathing disorders (SBD), that include snoring, obstructive sleep apnea syndrome (OSAS, a disorder in which a person often stops breathing during his or her sleep due to an obstruction of the upper airway) and the obesity-hypoventilation syndrome, defined as the combined presence of obesity (body mass index, (BMI) > 30 kg/m^2^) with awake arterial hypercapnia (PaCO_2_ > 45 mmHg) in the absence of other causes of hypoventilation [9], increase the risk of cardio- or cerebrovascular co-morbidity and mortality [10]. A high incidence of co-morbid medical conditions was also found for narcolepsy, a disorder of unknown etiology that is characterized by excessive sleepiness that typically is associated with cataplexy and other rapid eye movement (REM) sleep phenomena, such as sleep paralysis and hypnagogic hallucinations, as well as for other sleep disorders (e.g., restless leg syndrome (RLS) 15%), OSAS (25%)), obesity, cognitive deficit, psychiatric disorders (depression and anxiety), chronic pain, gastrointestinal disorders, hypercholesterolemia, and blood hypertension [11,12,13,14,15,16]. Patients with moderate to severe RLS report an increased incidence of sleep-related problems (up to 2–5 fold) [17,18], while severe RLS is frequently associated with depression, anxiety, obesity, OSAS, cardiovascular disorders, diabetes, erectile dysfunction, and end-stage renal disorders [19]. Research on twins has shown that sleep disorders often co-occur in a family in association with emotional, behavioral and health-related problems resulting from environmental rather than genetic factors [20]. Genetic factors nevertheless appear to account for the prevalence of sleep disorders in the evening or morning (80% of the phenotypic correlations). Major psychopathological symptoms and insomnia seem correlated with a shorter allele variant of the serotonin gene transporter region 5HTTLPR [21,22,23]. 

### 2.2. Sleep and Cardiovascular Disorders

Sleep disorders are a major risk factor for cardiovascular diseases [24]. A cross-correlation between insomnia, depression, and cardiovascular disorders has been reported in longitudinal studies [25,26,27]; recent evidence suggests that insomnia is associated with cardiovascular disorders also after adjusting for depression [28]. Persistent insomnia is associated with an increased risk of all-cause and cardiopulmonary mortality [29]. Difficulty in initiating sleep has been associated with myocardial infarction or coronary death in women, and constitutes a higher risk factor for cardiovascular disorders in females than in males (1.4 vs. 1.3) [30]. Altered circadian rhythms due to delayed sleep are relevant in the pathogenesis of cardiovascular diseases [27]. Insufficient sleep duration and poor sleep quality double the risk of hypertension and adverse cardio-metabolic effects [31]. Epidemiological studies show a significant association between insomnia and cardiovascular markers such as carotid intima-media thickness, cardiorespiratory fitness, and the Framingham risk score [32]. 

Moderate or severe, but not mild OSAS, increases the risk of cardiovascular disorders through a variety of mechanisms including intermittent hypoxia, chronic sympathetic activation, and systemic inflammation [33,34]. The duration of sleep with O_2_ saturation lower than 90% and other markers of inadequate sleep function (number of awakenings, mean heart rate, periodic leg movements, decreased total sleep time or excessive daytime sleepiness) increase the risk of developing cardiovascular disorders (5%–50%) by inducing endothelial dysfunction and sympathetic activation. OSAS may cause subclinical myocardial injuries, thus increasing the risk of heart failure. OSAS severity has been associated with higher levels of high-sensitivity troponin T in middle-aged and elderly subjects [35]. Treatment with continuous positive airway pressure (CPAP) may significantly reduce the occurrence of cardiovascular events; however, patients with poor oxygen desaturation at night and insufficient CPAP treatment (shorter than four hours per night) have a higher rate of hypertension or cardiovascular events than controls [36]. OSAS enhances the sympathetic tone and may induce hypertension, which in turn increases the risk of stroke. History or evidence of OSAS is frequent in patients with brain stroke or transient ischemic attacks and a dose–response relationship between OSAS severity and poor outcome after stroke has been reported [37,38]. Nocturnal hypoxemia is thought to be the major cause of health problems [39,40]. Surprisingly, however, CPAP treatment of these patients proved ineffective in preventing stroke in a randomized controlled trial [41]. 

### 2.3. Sleep Disorders and Diabetes

The association of cardiovascular disorders, diabetes and overweight/obesity is now collectively known as cardio-metabolic disorder. Risk factors include low socio-economic status, inadequate diet, sedentary lifestyles, and decreased duration and/or poor quality of sleep.

Glucose metabolism is regulated by the circadian system. Glucose distribution in the body is organized by a molecular clock in the liver [42], and metabolic processes are triggered and timed by food intake. Peripheral oscillator activity that regulates circadian cycles in peripheral tissues, controls temporal processes in the liver and pancreas. Liver nuclear receptors and adipocytes exhibit a 24 h periodicity. Temporal misalignment between the circadian period of adipocytes (peripheral oscillator) and the sleep/wake cycle (suprachiasmatic nucleus) plays a central role in reducing glucose tolerance and increases the risk of developing type-2 diabetes (T2DM) [43]. In a circular interaction, impaired metabolism promotes low expression of clock genes, while impaired clock timing disrupts metabolic activities, alters adipogenesis, favors obesity, and affects insulin sensitivity. Disrupted circadian mechanisms play an unequivocal role in abnormal adipogenesis, low glucose tolerance, impaired insulin responsiveness, and genetic susceptibility, which, in turn, are aggravated by excessive (high caloric) food intake and sedentary lifestyles [44,45]. Poor sleep has been associated with changes in appetite-regulating hormones (including lower levels of leptin and increased levels of ghrelin), increased subjective appetite and caloric intake, obesity or high BMI, or increased blood pressure [46,47]. 

Environmental factors disrupting the sleep/wake cycle may also affect metabolism indirectly. Working in shifts, insufficient exposure to sunlight, sleep disturbances, habitual eating late at night, and nocturnal exposure to artificial light are known to disrupt the circadian clock. Light exposure at night alters food timing and body mass accumulation in mice and is a potential contributing factor to the increasing prevalence of metabolic disorders [48]. Surveys have reported an increased prevalence of T2DM among night shift workers or subjects with irregular shift schedules and with exposure to light at night resulting in the disruption of sleep patterns and other markers of circadian synchronization [49]. 

### 2.4. Sleep Disorders and Psychiatric Conditions

Sleep problems are common in psychiatric disorders, worsen the outcome and may persist when the psychiatric condition has been successfully controlled by treatment [50]. About 90% of depressed patients complain about sleep disorders, which precede the outset of depression in 40% of cases [51]. A history of persistent insomnia is associated with an increased risk of developing a new depressive episode [52]. In a meta-analysis [53], a twofold higher risk was correctly predicted for clinically non-depressed subjects with insomnia compared to controls. Sleep disturbance is a core feature also in bipolar disorder, with insomnia hovering close to 100% and hypersomnia ranging from 23% to 78% during the depressive phase. As in unipolar depression, these patients experience shortened latency of REM sleep compared to controls [54], in particular during periods of mania when 69%–99% of subjects also report a reduced need of sleep. Depressive symptoms associated with insomnia have a greater negative impact on the quality of life than sleep disorders alone [55]. Sleep disorders are thought to contribute to developing depression via their effect on hippocampal functions; sleep restrictions enhance neuronal sensitivity to excitotoxic insults and vulnerability to neurotoxic challenges to a final decreased gray matter volume in hippocampus and in the left orbitofrontal cortex [56,57]. There is evidence that the association between disrupted sleep and depression is caused by a genetic background in which overlapping occurs between the genes influencing sleep disorders and depression and those affecting sleep disorders and anxiety (58% and 74%, respectively) [58,59]. Sleep disorders are prevalent during both active psychosis and remission in schizophrenia. Complaints are mostly about difficulties in falling asleep, awakenings during the night or early in the morning, poor sleep, or increased time in bed. These disturbances have been associated with severe symptoms in schizophrenia, particularly with positive symptoms such as delusion, hallucinations, confused thinking and behavior. Withdrawal symptoms such as anhedonia, social withdrawal, loss of motivation, or poor self-care are not related to insomnia [54]. 

### 2.5. Sleep Disorders, Behavior and Cognitive Impairment

Excessive drowsiness, changes in mood and impaired attention, memory and visuoconstructive functions are common in OSAS and SBD. These disorders may unfavorably interfere with the quality of life [60,61,62,63]. Working memory is impaired in OSAS because of both executive and attentional deficits [64,65,66]. About half of OSAS patients manifest a reduction in the information processing speed compared to controls, with a correlation between speed reduction and severity of disorder [64]. Hypoxemia and hypercapnia impair the ability to execute tasks regulated by frontal brain structures. CPAP treatments of moderate to severe OSAS result in a minor improvement in the processing speed when compared to conservative treatment and improve attention and vigilance, whereas the effects on memory are inconsistent and impairment in executive and visuoconstructive functions may persist [62,64,67,68,69]. It has been suggested that CPAP treatment is insufficient in these conditions [70]. 

Impaired attention and episodic memory is reportedly frequent among insomniacs [71,72]. Insomnia due to RLS impairs the ability to focus, memorize and perform tasks both at work or at school, due to alterations in prefrontal cognitive tasks and executive functions [73]. Cognitive impairment, reduced alertness, poor emotional adaptation, excessive drowsiness, social problems, etc. are also reportedly related to RLS [74,75]. Sleep deprivation also increases irritability and hyperactivity. It should be noted that sleepiness, depression and psychiatric co-morbidity rather than objective cognitive deficits may play a role in the subjective perception of attentional impairment [76]. In the elderly, insomnia and resulting sleepiness during the day increase the risk of accidental injury and may contribute to worsening cognitive impairment, depression, and metabolic syndrome [77]. 

Brain function is much more vulnerable to sleep loss in the morning than in the evening. Performance at any given time of the day depends on the interaction between the length of preceding wake episodes (homeostatic factor), individual chronic sleep debt, and the circadian phase (circadian factor) in which performance is assessed. Circadian variability in performance is more evident in the presence of sleep loss [78].

### 2.6. Mortality

Sleep disorders with predominant insomnia currently constitute risk factors for mortality, especially in males [29]. Two meta-analyses report an increased risk of mortality among short duration sleepers (about 10% of physiological sleeping time) and subjects who sleep for an excessive period of time (over 20%–30%) [79,80], but the evidence for a U-shaped association between sleep duration and mortality has been questioned [81]. 

Available data suggest a relationship between OSAS, all-cause mortality and cardiovascular pathological events as well as between mortality (all-cause or from cardiovascular disorders) and excessive daytime sleepiness [34]. In recent years, the possibility that changes in sleep duration and architecture may initiate, worsen or modulate the phenotypic expression of multiple diseases including cancer has gained increasing attention. Furthermore, intermittent hypoxia, which is correlated to sleep disordered breathing (SDB), has been implicated in higher incidence and a more adverse prognosis of cancer [82]. In a representative population, the adjusted risk of pancreatic and kidney cancer and melanoma were significantly higher in patients with OSAS [83]. An association between OSAS and breast [84] or central nervous system cancer [85] has also been hypothesized. Moderate to severe OSAS is regarded as an independent risk factor for cancer mortality [86]. However, owing to the methodological limitations of many of these recent studies on cancer, which are mostly retrospective and often lack any measurement of sleep fragmentation or hypoxia, new controlled, prospective studies are needed [87]. 

Narcolepsy is also associated with a 1.5 fold increase in all-cause mortality in the U.S. population. Excessive daytime sleepiness due to disordered sleep is recognized as a major health hazard, irrespective of other risk factors such as unhealthy lifestyles or co-morbidities such as depression [27]. This risk peaks in the 25–34 year (possibly due to the association of disordered sleep and depression) and 35–44 year age ranges [16].

Both insufficient and excessive sleeping are thought to increase the mortality risk due to co-morbidity [88]. Insufficient sleep is thought to increase the risk through adverse endocrine, immunologic, or metabolic effects, induction of chronic low-grade inflammation, increased cortisol secretion, or altered growth hormone metabolism [81].

On the contrary, the correlation between excessive sleep and diabetes, hypertension or cardiovascular diseases and a higher mortality risk remains undocumented [88,89]. 

### 2.7. Sleep Disorders and Quality of Life

There is a strong association across all economic, social and family categories and age ranges between disordered sleep and markers of life quality (poor status, family or social problems related to separation or divorce, unhealthy lifestyles, etc.). The subjective stress level is associated with complaints of poor sleep [90]. Socioeconomic inequalities explain a large portion of the gender-related differences in sleep problems [91]. 

Chronic insomnia is still associated with life quality after compensating for co-morbidities [2,92,93,94,95]. The effects of insomnia on life quality are age-related [96,97]. RLS symptoms and other sleep complaints are also associated with higher disability or lower physical function scores [75,98]. 

Narcolepsy has a substantial economic burden due to high healthcare costs and reduced income that is already evident years before diagnosis [88,99,100]; improvement over time in the health-related quality of life has also been reported [101] as a possible result of adaptation strategies developed to cope with the disease [16]. Excessive drowsiness during the day and alterations in circadian rhythms also affect personal or professional skills, health and subjective wellbeing [102]. Severe OSAS, which is the most common cause of excessive sleepiness and impaired alertness, affects the quality of life also by impairing the efficiency and quality of sleep in a circular process, especially if associated with obesity [103,104]. 

### 2.8. Sleep Disorders, Efficiency and Accidents at Work

There is evidence that absence from work, frequency of accidents, productivity, progression in career and professional reward are all worse in poor sleepers. Reduced efficiency at work is a costly burden on workers, healthcare and welfare programs, and employers. The working schedule and occupational requirements often reinforce or precipitate insomnia. Reduced efficiency at work and absenteeism due to insomnia increase over time. The relationship between efficiency at work and sleep quality is reciprocal; co-morbidities, self-reported poor efficiency at work and claims for earlier retirement because of acquired disability are more frequent in poor sleepers [105,106,107,108,109,110]. The prevalence of complaints about insomnia, poor sleep, and daytime sleepiness is higher in shift-workers than among those working on regular schedules [111,112,113]; in turn, sleep disorders reduce adaptation to shift schedules and increase the occurrence of accidents at work. 

Occupational injuries are a major problem worldwide. All markers of disordered sleep are associated with an increased risk of injuries at work and approximately 13% of injuries can be attributed to sleep problems [3]. Workers with sleep problems have a 1.62 fold higher risk of being injured at work than workers without sleep problems [114,115,116,117,118]. The extent to which the risk of accident is influenced by the use of hypnotics is unclear; self-medication is deemed frequent, but to date remains poorly documented [105]. The loss in overall professional efficiency among people suffering from sleep disorders has been estimated to be about 10.3% higher than among good sleepers [113,119]. The National Sleep Foundation survey (2008) found the risk of accidents and injuries at work higher among workers reporting sleep latency longer than 30 min [120]. 

### 2.9. Sleep Disorders and Road Safety

Casualties in motor vehicle accidents total over 3000 per day around the world, with over 1.3 million deaths per year; an additional 20 to 50 million people are injured and often remain disabled for life [121]. According to the European _Statistics on Accidents at Work (ESAW) [122], road accidents accounted for 9.6% of all accidents at work in 2007. Human errors are blamed in 90% of road accidents [123], the main human factors being fatigue and sleepiness [115,124,125].

Insomnia, OSAS, hypersomnias, and sleepiness during the day increase two to seven fold the risk of traffic accidents among professional drivers [126,127,128,129,130,131,132,133,134,135,136,137,138,139,140,141]. The accident risk is increased in both non-professional [125,142,143] and professional drivers with OSAS [139,144,145,146,147]. In an influential study comparing OSAS and controls [148], apneic drivers with an apnea-hypopnea index ≥ 10 had a 6.3 (95% confidence interval (CI): 2.40–16.02) odd ratio probability of being involved in a traffic accident. This relationship remained stable even after adjustment for alcohol, visual problems, BMI, driver’s age and seniority, history of traffic accidents, medications causing drowsiness, and sleep schedule. Insufficient evidence has prevented an assessment of the risk of road accidents caused by narcolepsy [137]; the incidence of sleepiness-related accidents is nevertheless reportedly high among narcoleptics [126], while impaired and unsteady cognitive performance has been observed in driving-simulation studies [147,148,149]. About 16% of subjects suffering from RLS or periodic limb movement have been involved in traffic accidents [126]. 

Complaints of severe daytime sleepiness and reports of near miss accidents due to sleepiness are correlated, particularly in OSAS [150,151]. In a cohort study, approximately 50% of drivers surviving a collision had at least one sleep-related risk factor [152]; about 16.9% of those interviewed reported a sleep disorder, 5.2% reported OSAS, 9.3% insomnia, and 0.1% narcolepsy and hypersomnia. About 8.9% of habitual highway drivers reported experiencing at least once a month sleepiness fits that were so severe they had to stop driving. One-third of interviewed drivers (31.1%) reported near miss accidents (50% of which were sleep-related), 7.2% reported a driving accident in the past year, and 5.8% of accidents were deemed sleep-related [153].

### 2.10. Driving Licenses and OSAS in E.U. Regulations

Since 2014, European legislation has established new driving license regulations for subjects with OSAS in an attempt to control risks related to sleep problems. The EU Commission (1 July 2014) [154] amended the previous directive 2006/126/EC by stating in Annex III that: “…*Applicants or drivers in whom a moderate or severe OSAS is suspected shall be referred for further authorised medical advice before a driving license is issued or renewed. They may be advised not to drive until confirmation of the diagnosis […]. Driving licenses may be issued to applicants or drivers with moderate or severe OSAS who show adequate control of their condition and compliance with appropriate treatment and improvement of sleepiness, if any, confirmed by authorised medical opinion […]. Applicants or drivers with moderate or severe OSAS under treatment shall be subject to a periodic medical review […], with a view to establish the level of compliance with the treatment, the need for continuing the treatment and continued good vigilance.*” This strong commitment on the part of the EU Commission is proof of an increasing awareness of the importance of OSAS in public health.

### 2.11. Direct/Indirect Economic Impact of Sleep Disorders

It is difficult or impossible to introduce comparable objective measures in different countries, but there is evidence of the high costs of sleep disorders. The Australian Sleep Health Foundation commissioned Deloitte Access Economics (a nation-based company) to analyze the direct and indirect costs of sleep disorders for the year 2010 and the reported estimates describe the magnitude of the problem [155]. The estimated costs associated with the three commonest sleep disorders (OSAS, primary insomnia, and RLS) were $818 million per year, including $274 million for sleep disorders and $544 million for associated conditions. About $248 million were the costs for OSAS and $409 million for related healthcare conditions such as hypertension, cardiovascular diseases, and depression. The indirect financial and non-financial costs were estimated to be above $4.3 billion, of which OSAS accounted for 61% ($2.6 billion), primary insomnia 36% ($1.5 billion) and RLS 3% ($115 million) [154]. Impaired quality of life due to sleep disorders and related conditions was estimated to a total non-financial cost of up to $31.4 billion. 

## 3. Conclusions

The disruption of physiological circadian rhythms resulting from sleep disorders or professional/voluntary sleep deprivation stands as a major risk factor for psychiatric, cardiovascular, metabolic or hormonal diseases and affects everyday life to a considerable extent. A significant association with impaired cognitive functions and professional efficiency, increased error rates and reduced safety at work or when driving has been reported in surveys and population-based studies. The direct and indirect costs of sleep disorders and poor sleep are high. 

Recently, health authorities have also shown more interest in sleep disorders. The efficacy of enforced countermeasures is yet to be proved and needs to be confirmed through monitoring and over a proper length of time. On the contrary, poor sleep is usually neglected. If the healthcare community receives ample and detailed information about this problem, procedures can be introduced to promote (partial) control of the negative effects of major sleep disorders. On the contrary, the negative effects of sleep deprivation (particularly if voluntary) remain outside the medical domain and are difficult to counterbalance. However, shift work can be regulated, so working schedules could therefore be designed to have a reduced impact on circadian rhythms. In fact, closer medical control, proper promotion of sleep hygiene and healthier lifestyles in shift-workers are desirable in order to continuously improve occupational health [156]. Protocols and procedures could be developed to identify and monitor vulnerable individual subjects or categories. 

Adequate strategies for preventing the medical and nonmedical effects of sleep disorders or sleep deprivation would help to reduce healthcare, welfare, personal and social costs and promote a better quality of life.
